# Design, Characterization, and Immune Augmentation of Docosahexaenoic Acid Nanovesicles as a Potential Delivery System for Recombinant HBsAg Protein

**DOI:** 10.3390/vaccines10060954

**Published:** 2022-06-16

**Authors:** Mohammed Ali Bakkari, Sivakumar S. Moni, Abdulrahman Alshammari, Ahmad Salawi, Muhammad H. Sultan, Osama A. Madkhali, Saad S. Alqahtani, Mohammad Firoz Alam, Emad Sayed Shaheen, Mohamed Eltaib Elmobark

**Affiliations:** 1 Department of Pharmaceutics, College of Pharmacy, Jazan University, Jazan 45142, Saudi Arabia; mbakkari@jazanu.edu.sa (M.A.B.); asalawi@jazanu.edu.sa (A.S.); mhsultan@jazanu.edu.sa (M.H.S.); omadkhali@jazanu.edu.sa (O.A.M.); mohamedeltaib.me@gmail.com (M.E.E.); 2 Department of Pharmacology and Toxicology, College of Pharmacy, King Saud University, Riyadh 11451, Saudi Arabia; abdalshammari@ksu.edu.sa; 3 Department of Pharmacy Practice, College of Pharmacy, Jazan University, Jazan 45142, Saudi Arabia; ssalqahtani@jazanu.edu.sa; 4 Pharmacy Practice Research Unit, College of Pharmacy, Jazan University, Jazan 45142, Saudi Arabia; 5 Department of Pharmacology and Toxicology, College of Pharmacy, Jazan University, Jazan 45142, Saudi Arabia; firozalam309@gmail.com; 6 Medical Research Centre, Jazan University, Jazan 45142, Saudi Arabia; emadshaheen@hotmail.com

**Keywords:** vaccine adjuvants, recombinant proteins, HBsAg, nanovesicles, docosahexaenoic acid

## Abstract

Recombinant HBsAg-loaded docosahexaenoic acid nanovesicles were successfully developed, lyophilized (LRPDNV) and characterized for their physico-chemical properties. The zetapotential (ZP) of LRPDNV was −60.4 ± 10.4 mV, and its polydispersity (PDI) was 0.201, with a % PDI of 74.8. The particle sizes of LRPDNV were 361.4 ± 48.24 z. d.nm and 298.8 ± 13.4 r.nm. The % mass (r.nm) of LRPDNV in a colloidal injectable system was 50, its mobility value was −3.417 µm cm/Vs, while the conductivity of the particles was 0.728 (mS/cm). Transmission electron microscopic (TEM) analysis showed smooth morphological characteristics of discrete spherical LRPDNV. Differential scanning calorimetry (DSC) and thermogravimetric analysis (TGA) of LRPDNV revealed that LRPDNV is thermostable. The X-ray diffraction (XRD) studies showed a discrete crystalline structure of LRPDNV at *2θ*. Nuclear magnet resonance (NMR) studies (1H-NMR and 13C-NMR spectrum showed the discrete structure of LRPDNV. The immunogenicity study was performed by antibody induction technique. The anti-HBs IgG levels were elevated in Wistar rats; the antibody induction was observed more in the product (LRPDNV) treatment group when compared to the standard vaccine group. The level of antibodies on the 14th and 30th day was 6.3 ± 0.78 U/mL and 9.24 ± 1.76 U/mL in the treatment and standard vaccine groups, respectively. Furthermore, the antibody level on the 30th day in the treatment group was 26.66 ± 0.77 U/mL, and in the standard vaccine group, the antibody level was 23.94 ± 1.62 U/mL. The LRPDNV vaccine delivery method released HBsAg sustainably from the 14th to the 30th day. The results of this study indicate the successful formulation of DHA nanovesicles which have great potential as an adjuvant system for the delivery of recombinant HBsAg protein.

## 1. Introduction

Recombinant proteins are widely used as biopharmaceutical products due to their demonstrated safety profile. Numerous recombinant proteins with therapeutic and preventive potential have been successfully generated in the modern period [1]. The hepatitis B virus surface antigen (HBsAg) is a highly immunogenic protein that, when administered as a vaccine, can elicit a favorable immunological response in the recipient. The use of recombinant DNA technology has made it possible to obtain large amounts of HBsAg. The current recombinant HBsAg vaccination program has resulted in a decrease in the incidence of the illness globally. However, there is no guarantee that immunity will last beyond the age of 40, while repeated vaccinations may result in seroconversions [2,3]. On the other hand, a multiple dosage regimen is experiencing a limited number of responders for follow-up vaccinations due to a lack of understanding about the need for booster doses. This lethargy is likely attributable to the global economic crisis or the sceptical nature of social and cultural ethos [4]. Many significant obstacles in designing effective vaccines for new diseases as well as improving the immune responses of currently available vaccines are considered big challenges [5]. The concept of vaccine-provoked immunity is highly dependent on the adjuvants used in the formulation of the vaccine. The selection of an appropriate adjuvant is the basic requirement for improving the efficacy of recombinant vaccines [6].

The latest adjuvant which has been approved for use in recombinant HBsAg is AS04 [7]. However, the vaccination schedule of recombinant HBsAg vaccine varies with the mode of transmission of disease [8]. Polymeric particle technology has not been very effective due to its high toxicity profile and the difficulty of targeting the innate and adaptive immune systems because of its biodegradability. Although highly cationic biodegradable polymers have been investigated over the last 25 years, this concept is still in its infancy. Fatty acids are amphiphilic single-chain molecules composed of a long non-polar carbon chain terminated by a polar carboxylic acid group. Fatty acids have played a critical part in biological operations and are believed to be present in a variety of bodily tissues and fluids; specifically, they are stored in the cytoplasm of cells and serve as a significant source of energy. Fatty acids have been widely used as adjuvant vehicles in drug delivery to improve the penetration of drugs at a cellular level [9]. Omega-3 fatty acids are highly beneficial to normal physiological function in humans, and it maintains good cellular health [10]. An omega-3 fatty acid vesicular formulation will have a high degree of membrane binding and will be extremely biocompatible with the cellular system because the skin contains omega-3 fatty acids as one of its components. Significant conceptual advancements in innate immunity and the elucidation of the crucial innate receptors, the pattern recognition receptor, have led to the understanding that numerous commonly used live vaccines cause immune responses by activating specific pattern recognition receptors. Therefore, the detection of innate immune cells will target the cytokine network to activate CD4+ and CD8+ cells [11].

Docosahexaenoic acid (DHA), an omega-3 fatty acid, is widely used to treat various human ailments, most notably heart disease and cancer. DHA has been reported to have membrane-bound actions via integrating into membrane phospholipids, hence increasing membrane permeability [12]. As a result, the purpose of this study was to develop a safe and effective adjuvant system for the naïve delivery of recombinant HBsAg, which is more significant for the induction of robust immune response. In the present study, recombinant HBsAg entrapped DHA nanovesicles were developed, then evaluated for adjuvant compatibility using physicochemical characterization and determining the antibody induction through in vivo immunogenicity study.

Earlier reports suggest that the DHA can inhibit proinflammatory cytokine networks [13]. In contrast, the DHA vaccine delivery system, on the other hand, can act as a depot reservoir to extend antigen release; because DHA is a component of the skin, it has membrane-bound effects by integrating into membrane-bound products and is biocompatible within the cellular system [14]. The present study demonstrates that DHA vesicular formulation-activated immune cells produce specific antibodies sustainedly. As a result, DHA nanovesicles can be used as a delivery system for recombinant HBsAg, allowing the HBsAg to deliver sustainedly in a naive form at the cellular level.

## 2. Materials and Methods

### 2.1. Materials

Recombinant HBsAg, 20 mcg/mL concentration adsorbed on 0.5 mg as aluminum hydroxide. Docosahexaenoic acid was purchased from Sigma, St. Louis, MO, USA. Syringe PVDF Filter Unit (0.2 µm) was purchased from Merck, Darmstadt, Germany. The solvents used in this study were products of Scharlau, Barcelona, Spain.

### 2.2. Formulation of DHA Nanovesicles

The nanovesicles were prepared using a simple film hydration method. A 1:1 proportion of methanol and DHA was subjected to stirring at a fixed rate of 2000 rpm in a clean, sterile glass beaker on a hot plate for 15 min, initially at 60 °C. Thereafter, 1 mL of 10% *v*/*v* (2 mcg) recombinant HBsAg sample was added to the mixture three times at predetermined time intervals to develop a reaction mixture (RM). The RM was sonicated for 5 min at full amplification. Then, it was placed on a rotary shaker at 200 rpm for 20 min. The same procedure was repeated twice to develop recombinant HBsAg-loaded DHA nanovesicles (RPDNV). The RPDNV was further kept in a heating mantle at 60 °C for 45 min to allow for the development of a thin film. Finally, the thin film was eluted in methanol and subjected to lyophilization and various physiochemical characterization procedures to ascertain the characteristics of the RPDNV.

### 2.3. Lyophilization Process

The RPDNV was lyophilized by freeze-drying using Millrock BT85 equipment (Millrock Technology, Kingston, NY, USA). A 1:1 volume ratio of 5% mannitol solution and RPDNV was prepared. The combination was deep-frozen at −80 °C for 24 h, following which the glass flask containing the mixture was placed in lyophilizing tubes, and the vacuum was induced by opening the knob. The vacuum pressure was at 3000 pascals at −84 °C. After 24 h of lyophilization, the lyophilized RPDNV (LRPDNV) crystals were eluted and kept at +4 °C until used in subsequent studies [15].

### 2.4. Preparation of Sample Analyte

A 1% (*w*/*v*) mixture of LRPDNV and Millipore water was placed on a hotplate and stirred using magnetic bead for 5 min at constant rotation (1000 rpm) to develop a consistent mixture which was then subjected to filtration using a Millex-GV Syringe PVDF Filter with 0.2 µm pore size. The liquid LRPDNV filtrate was subjected to the following analyses:

### 2.5. Physicochemical Characterization

#### 2.5.1. Dynamic Light Scattering (DLS) Analysis

Surface charge, vesicle size, and polydispersity index (PDI) were essential parameters used to physically characterize LRPDNV in an injectable dosage form. The surface charge was determined with zeta potential (ZP) analysis, while LRPDNV nano-size (NS) and PDI in the injectable colloidal system were measured with the DLS procedure. These parameters were estimated with a Malvern Nano-ZS Zetasizer (Malvern Panalytical, Malvern, UK). The filtrate of LRPDNV was put in a capillary cell devoid of air bubbles prior to positioning in the Zetasizer sample chamber. The test was performed using a standard procedure in accordance with the manual guidelines provided by Malvern Instruments, UK.

#### 2.5.2. Transmission Electron Microscopy (TEM)

Crystal and liquid samples of LRPDNV were subjected to TEM in line with the procedure described previously [13]. TEM is a technique that results in a very high resolution of images of nanoparticles. A powder sample and liquid sample of LRPDNV were characterized using JEOL JEM-1011 transmission electron microscope (JEOL Ltd., Tokyo, Japan). The TEM grid was prepared by placing the sample on a carbon-coated grid, and the instrument was operated at 200 kV. The TEM procedure was carried out in line with the method reported earlier.

#### 2.5.3. Differential Scanning Calorimetry (DSC)

The lyophilized LRPDNV crystals were subjected to DSC using Shimadzu DSC 60 (Shimadzu, Kyoto, Japan) in line with the procedure reported previously [16]. The powdered LRPDNV sample was placed in aluminum pans that were not hermetically sealed. The temperature was increased from 30 to 350 degrees Celsius at a rate of 10 °C per minute, while the atmospheric airflow was maintained at 10 mL per minute.

#### 2.5.4. Thermogravimetric Analysis (TGA)

The thermal stability of the samples in an air atmosphere was assessed using a thermogravimetric analyzer (TGA 8000, Perkin Elmer, Waltham, MA, USA). The lyophilized LRPDNV powder sample (10 mg) was placed on an aluminum pan, and the test was conducted at a heating rate of 10 °C/min within a temperature range of 50–300 °C.

#### 2.5.5. X-ray Diffraction (XRD) Analysis

The LRPDNV crystals were subjected to structural analysis using XRD in accordance with a previously reported procedure [17]. The crystalline structure was used as an index of purity of LRPDNV. The lyophilized powder sample was subjected to XRD using a Unisantis XMD 300 X-ray powder diffractometer (Unisantis Europe GmbH, Georgsmarienhutte, Germany). The XRD diffractograms were obtained at *2θ* in the range 2–50° using Cu K α radiation of incident beam (λ = 1.5418 Å) at a voltage of 45 kV and a current of 0.8 mA. A scanning range of *2θ*/*θ* was selected and scanning speed of 10 min^−1^ was employed.

#### 2.5.6. Nuclear Magnetic Resonance (NMR) Spectroscopy

The LRPDNV was subjected to nuclear magnetic resonance (NMR) spectral analysis. Samples were previously prepared in deuterated water (D_2_O). The NMR spectrum of the crystal sample was recorded on Bruker 400 Ultra shield NMR spectrometer operating at 400 MHz to obtain ^1^H-NMR and at 100 MHz for ^13^C NMR in deuterated chloroform (DCCl_3_) solvent with tetramethyl silane (TMS) as an internal standard.

#### 2.5.7. Loading Studies

One (1.0) g of LRPDNV was placed in 10 mL of extracting medium that contained phosphate-buffered saline (PBS), pH 7.4 and 0.1N HCl in a proportion of 1:1. The mixture was placed on a hot plate with a magnetic bead for 30 min at room temperature. Thereafter, the mixture was centrifuged at 2000 rpm for 15 min. The supernatant was collected, and the loading was determined using an enzyme-linked immunosorbent assay with HBsAg one, version ultra (DIA PRO, Milano, Italy). Then, the percentage recombinant HBsAg loading (VL) was calculated using the following Equation:

(1) $$\mathrm{VL}\;(\%)=\frac{\mathrm{Total}\;\mathrm{amount}\;\mathrm{of}\;\mathrm{recombinant}\;\mathrm{HBsAg}\;\mathrm{incorporated}\;\mathrm{in}\;\mathrm{LRPDNV}\;\times 100}{\mathrm{Total}\;\mathrm{weight}\;\mathrm{of}\;\mathrm{LRPDNV}}$$

#### 2.5.8. In Vitro Release Profile

Exactly 500 mg of LRPDNV was dialyzed in 50 mL of phosphate-buffered saline (154 mM, pH 7.4) containing 250 µL of Tween 80. The dialysis system was kept on a rotary shaker which was rotated at a speed of 200 rpm. The temperature was maintained at 37 °C and the experiment was performed for a duration of 8 h. The samples were analyzed at 0, 1, 2, 3, 4, 5, 6, 7, and 8 h by performing enzyme-linked immunosorbent assay using HBsAg one, version ultra (DIA PRO, Milano, Italy). The release profile was calculated by extrapolating the test values from a standard curve prepared by plotting OD against HBsAg concentration.

### 2.6. In Vivo Study

#### 2.6.1. Preparation of Test Sample

A 1% *w*/*v* of the test sample (Product) was prepared by mixing a specified quantity of LRPDNV with a known volume phosphate buffer of pH 7.4. The mixture was maintained on a hotplate for 10 min at room temperature while being stirred with a magnetic stirrer bead. Then after, the product was filtered using a syringe PVDF filter unit (0.2 µm). The filtrate was used to immunize the Wistar rats to determine specific IgG antibody induction.

#### 2.6.2. Immunogenicity Profile

Immunogenicity studies were carried out by antibody induction method [8] on healthy male Wistar rats weighing about 150–200 g. The animals were maintained and handled according to the standard guidelines. The animals were acclimatized under standard protocols: the temperature was maintained, and the humidity was about 56 ± 6%, achieved by exposure with an alternating 12 h light/dark cycle. The animals had access to fresh water and fed with standard diet pellets. Animal studies were conducted in accordance with the recommendations of the Institutional Animal Ethical Committee. Institutional Animal Ethical Committee, College of Pharmacy, Jazan University, Jazan, KSA, authorized the experimental protocols. The animals were separated into four groups, each containing six rats and the protocol as follows:

**Group 1:** Normal group—the animals did not receive any LRPDNV or vehicles.

**Group 2:** Product treatment group—the animals were immunized with 0.5 mL (equivalent to 1 mcg) of LRPDNV intra peritoneally.

**Group 3:** Vehicle treatment group—the animals were immunized with 0.5 mL of lyophilized nanovesicles intra peritoneally.

**Group 4**: Standard vaccine treatment group—the animals were immunized with 0.5 mL of marketed HBsAg (1 mcg) vaccine intra peritoneally.

The duration of immunogenicity study was 30 days; the animals of group 2, 3, and 4 received a booster dose on 14th day with the same test samples. Blood samples were collected from the retroorbital plexus using a capillary tube on the 16th and 30th days. Sera were separated by centrifugation and stored at −20 °C until assayed.

#### 2.6.3. Determination of Antibodies to HBsAg

The immunopotentiation was measured by quantifying specific antibodies with a rat anti-HBsAg IgG enzyme-linked immunosorbent test kit (catalogue number 4270) from Alpha diagnostic International, United States. The test is based on the binding of rat anti-HBsAg in the serum samples to HBsAg immobilized on microwells of a microtiter plate. By the addition of horse radish peroxidase enzyme, the substrate tetramethyl benzidine (TMB), and stopping solution. The intensity of the color developed was measured by determining its absorbance at 450 nm using a BioTek ELx 800 ELISA reader (BioTek, Winooski, VT, USA). The concentration of anti-HBsAg was determined by extrapolating on the standard curve.

#### 2.6.4. Preparation and Validation of Standard Cure

The working standard solutions of concentrations 120, 100, 80, 60, 40, and 20 U/mL were prepared via serial dilution of the positive control solution of the kit in Millipore water. The calibration curve was created by measuring the at 450 nm in a UV/visible spectrophotometer. The method was validated by determining linearity at specific wavelengths, which indicates consistency with Beer–Lambert’s law. The standard curve was prepared by plotting the absorbance values at λmax against anti-HBsAg IgG concentration.

### 2.7. Histopathological Analysis

Histopathological analysis was carried out as per Alam et al. 2018 [17]. In brief, the liver tissue of each group of rats was isolated and fixed in 10% *v*/*v* formalin solution for histopathological analysis. Cross-sections (4–5 µm thick) of the fixed tissues were produced by microtome (Leica PM 2125, Germany) and stained in hematoxylin and eosin (H&E) for histological analysis through a microscope. The cross-sections were visualized under a light microscope (Nikon, Tokyo, Japan) to study the microscopic architecture of the liver.

### 2.8. Statistics

Data processing was undertaken with GraphPad Prism 9 (GraphPad Software, San Diego, CA, USA). Comparison amongst groups was carried out with ANOVA and Tukey’s post hoc test. Statistical significance was fixed at *p*  <  0.05.

## 3. Results and Discussion 

The concept of a vaccine delivery system emerged 30 years ago. It was evolved from polymeric microspheres, and it has progressed to polymeric nanoparticle technology. However, to date, the significance of the research work is not fully accomplished because of the burst release of vaccine from the polymeric particulate delivery system, non-predictable dosage forms, and the problem of toxicity at a cellular level, which is an unpleasant property, although some nanoparticles are highly biocompatible and biodegradable. Thus, an ideal vaccine adjuvant should be a two-sided blunt knife, i.e., it should neither affect the vaccine antigen nor should it produce any adverse effects after administration to the human individuals. In this connection, omega-3 fatty acids are highly beneficial to the normal human physiological function, and they maintain good cellular health. The purpose of this work was to develop a novel docosahexaenoic acid (DHA) nano vesicular system as an adjuvant for sustained delivery of recombinant HBsAg. Aiming to be a more effective adjuvant in vaccine formulation than currently used adjuvants through physicochemical characterization.

### 3.1. Physical Characterization of LRPDNV

#### 3.1.1. Dynamic Light Scattering (DLS) Analysis

**Figure 1:**
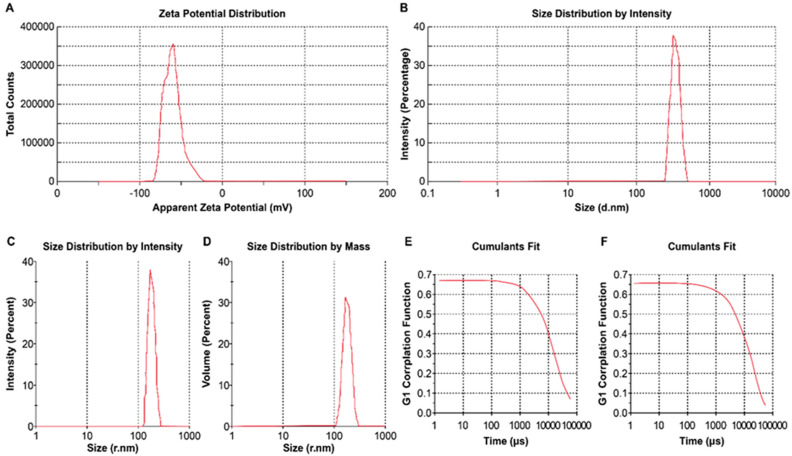
The physical characterization of lyophilized recombinant HBsAg–loaded docosahexaenoic acid nanovesicles. (**A**) Zeta potential of nanovesicles in the injectable formulation; (**B**) size distribution analysis by intensity in d. nm in the injectable formulation; (**C**) size distribution analysis in r.nm in the injectable formulation; (**D**) size distribution analysis by mass in the injectable formulation; (**E**) cumulative fit of particle size in an injectable formulation (**F**) distribution fit analysis of particles in the injectable formulation.

The lyophilized recombinant HBsAg-loaded DHA nanovesicles (LRPDNV) were successfully formulated as a crystalline complex that flowed freely. Table 1 shows that the physical properties of LRPDNV were good. In the present investigation, the injectable LRPDNV produced ZP in a single peak, thereby indicating uniform zeta potential (Figure 1A). The ZP of LRPDNV was −60.4 ± 10.4 mV, indicating that the formulation was highly stable. It has been suggested that ZP values greater than or equal to 25 mV are associated with high degrees of stability [18]. These values are generally regarded as having sufficient repulsive forces to achieve better physical colloidal stability [19]. The results revealed that the polydispersity index (PDI) of the injectable LRPDNV formulation was 0.201, with a % PDI of 94.8, indicating that the injectable vaccine formulation was highly homogenous. An earlier study found that injectable DSS nanoparticles had a PDI value of 0.6 and a % PDI of 77.8, indicating the creation of a homogeneous system [15].

The size distribution characterization of LRPDNV through intensity showed a uniform formulation represented by a unique peak (Figure 1B). The observed nanovesicle size was 361.4 ± 48.24 z.d.nm. The size of the injectable LRPDNV was also expressed in radius, i.e., 298.8 ± 13.4 r.nm, and its distribution through the intensity peak revealed uniform particle distribution (Figure 1C). The size distribution of injectable LRPDNV is presented in Figure 1D. The % mass (r.nm) value was 100; this is a good indication that the size LRPDNV was uniformly distributed in the injectable dosage form. The size of the particle is very important for passive diffusion across the tissue barriers from the site of injection to the general circulation [20]. Indeed, nanoparticle size dictates the rate of cellular absorption. According to an earlier report, nanoparticles of sizes 50 to 200 nm are ideal for cellular uptake for cancer cell targeting [21]. Interestingly, a recent study found 100% internalization of nanoparticles with diameters ranging from 300 to 400 nm in macrophages when compared to lower sizes of nanoparticles [22]. Therefore, the present formulation is ideal for the uptake of innate immune cells. The sizes of particles influence the mobility of the particles in a colloidal system. Size is also an important factor that influences conductivity. Studies have suggested that particle sizes less than <20 nm have high mobilities that influence particle surface charge. In this study, the particle sizes ranged from 300 to 400 nm, which showed less mobility, thereby indicating more sustainability [23,24]. Based on cumulative and distribution fit analyses of particles, the LRPDNV formulation was of high quality in a colloidal injectable formulation, with 100% linearity (Figure 1E,F).

#### 3.1.2. Morphological Analysis of LRPDNV

**Figure 2:**
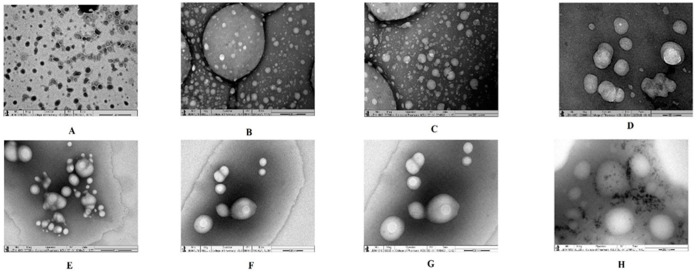
Transmission electron microscope study of nanovesicles. (**A**) Lyophilized recombinant HBsAg loaded docosahexaenoic acid nanovesicles at ×6000 magnification; (**B**) lyophilized recombinant HBsAg loaded docosahexaenoic acid nanovesicles at ×50,000 magnification; (**C**) lyophilized recombinant HBsAg loaded docosahexaenoic acid nanovesicles at ×80,000 magnification; (**D**) lyophilized recombinant HBsAg loaded docosahexaenoic acid nanovesicles at ×200,000 magnification; (**E**) liquid form of recombinant HBsAg loaded docosahexaenoic acid nanovesicles at ×30,000 magnification; (**F**) liquid form of recombinant HBsAg loaded docosahexaenoic acid nanovesicles at ×40,000 magnification; (**G**) liquid form of recombinant HBsAg loaded docosahexaenoic acid nanovesicles at ×50,000 magnification; (**H**) liquid form of recombinant HBsAg loaded docosahexaenoic acid nanovesicles at ×80,000 magnification.

Analysis using TEM was performed for both liquid and lyophilized samples. The results of TEM analysis are presented in Figure 2 at various magnifications. Figure 2A presents the lyophilized nanovesicles (LRPDNV) at ×6000 magnification. The vesicles were discrete, with most of them being spherical, while a few were irregular in shape. They were more prominent in spherical shape when viewed at higher magnifications, i.e., at ×50,000 and ×80,000 (Figure 2B,C). However, both vesicles had spherical and smooth surfaces. In contrast, a few vesicles were non-homogenous and bigger in size, while some were aggregated. This might be due to the lyophilization process. In an earlier study, the DSS nanoparticles were aggregated due to the lyophilization process [15]. Interestingly, the vesicles showed outer and inner layers (Figure 2D) when viewed at a magnification of ×200,000. The surface of the vesicles was smooth, and some vesicles were irregular in shape, which might be due to lyophilization since a similar feature was not observed when the vesicles were analyzed as a liquid formulation. Figure 2E–H show the results of TEM analysis of liquid samples of nanovesicles (RPDNV). The RPDNV were smooth-surfaced, discrete, and spherically shaped bi-layered vesicles at a magnification of ×30,000, and only a few vesicles were aggregated. At magnifications of ×40,000 and ×50,000, the RPDNV was more discrete, with smooth and bilayer morphological features. It is noteworthy that at ×80,000 magnification, RPDNV became unique, with a spherical, smooth texture, were denser inside the vesicles than outside, and had a more discrete morphology. These observations in TEM analysis demonstrated that the nanovesicles were unilamellar, and a few trapped vesicles were observed in both lyophilized and non-lyophilized formulations. A similar type of finding was recorded in cryo-TEM analysis of DHA vesicles [12]. Therefore, from the present study, it is obvious that the nanovesicles were successfully developed. The lyophilization process affected the vesicle morphology to a small extent, as reported here. However, to the best of our knowledge, a similar type of work has not been reported before now.

#### 3.1.3. Thermal Analysis

**Figure 3:**
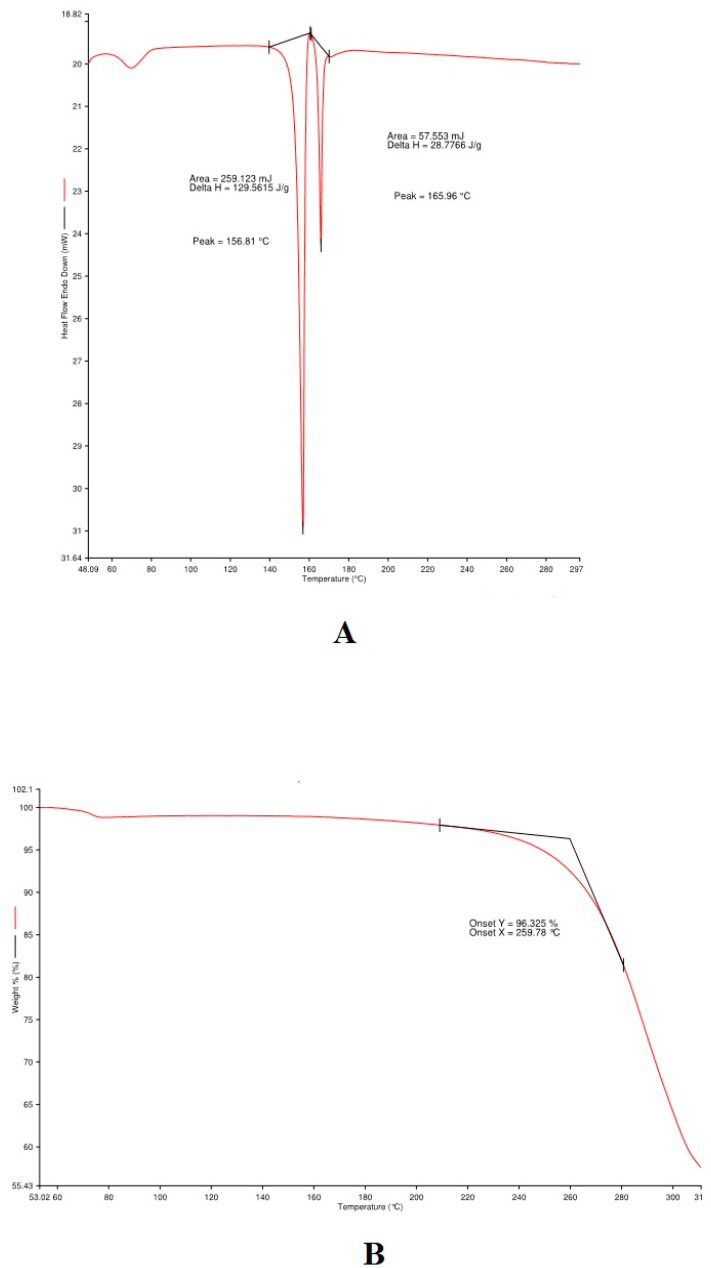
Physical characterization of lyophilized recombinant HBsAg-loaded docosahexaenoic acid nanovesicles; (**A**) differential scanning calorimetry study; (**B**) thermo gravity analysis.

Thermal experiments were used to investigate the thermal stability of LRPDNV using DSC and TGA. The former (DSC) is a thermal analysis technique that determines how much energy a sample absorbs or emits as a function of temperature. Physical variables, chemical interactions, and phase transitions cause variations in thermodynamic indexes of nanovesicles such as enthalpy, entropy, and heat capacity. These changes are usually measured with DSC. The endothermic behavioral curve of LRPDNV is depicted in Figure 3A. A short endothermic peak was observed at 70 °C, followed by a sharp endothermic peak which was observed at 156.81 °C with a ΔH value of 129.5615 J/g, which was followed by another sharp endothermic peak at 165.96 °C with a ΔH value of 28.7766 J/g. An earlier study reported the thermal degradation property of DHA during the deodorization of fish oil. The study showed that the degradation of DHA was observed at 180 °C [25]. The present study showed that LRPDNV degraded thermally from 156 to 165.34 °C, but the nanovesicles remained stable up to 156 °C, although a little degradation was observed at 70 °C. This indicates that a glass transition (Tg) temperature was attained above 156 °C, which resulted in molecular weight changes due to degradation. The thermal stability of the nanovesicles in the presence of oxygen was measured using TGA analysis. Figure 3B depicts the results of the TGA analysis of LRPDNV, showing a unique peak consistent with a degradation effect. Interestingly, from the thermogram, it can be understood that the initial weight loss in LRPDNV was observed around 70 °C. At 259.78 °C, about 96.3% weight loss was observed, indicating that LRPDNV was thermo unstable at high temperatures. An earlier study demonstrated that the eicosapentaenoic (EPA) rich oil was thermo-stable up to 150 °C, with weight loss of 38% between 150 to 300 °C. Interestingly the study also demonstrated that DHA-enriched oil showed increased thermal stability, when compared to EPA-rich oil, with the DHA-enriched oil showing total weight loss between 215 °C and 600 °C [26]. Interestingly the study revealed 15% weight loss in DHA-loaded microparticles at 110 °C because of humidity adsorbed in microparticles. In a prior study, TGA analysis of recombinant HBsAg encapsulated chitosan (CS) and mannosylated chitosan (MCH) nanoparticles revealed weight loss in two stages, resulting in the suggestion of two decomposition phases. At temperatures lower than 150 °C, the initial stage of weight loss for CS and MCH occurred due to water evaporation. The second weight loss for CS began at a temperature of about 215 °C and persisted until it reached 900 °C because of chitosan. The present study has demonstrated the thermostability of LRPDNV, and its stability and suitability in an injectable dosage form.

#### 3.1.4. XRD Analysis

**Figure 4:**
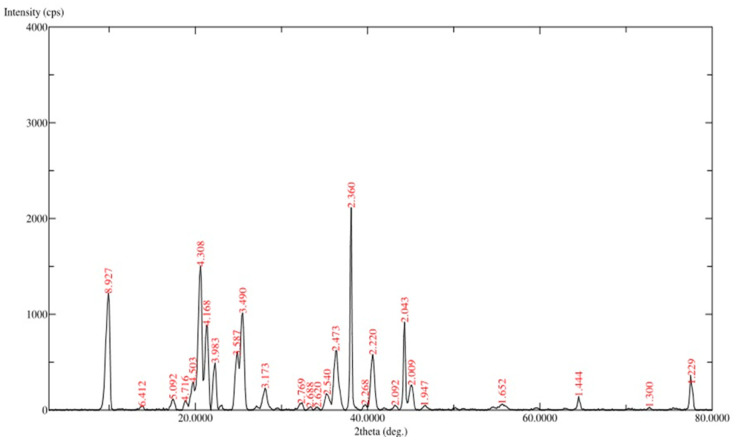
XRD analysis of lyophilized recombinant HBsAg-loaded docosahexaenoic acid nanovesicles.

Results from XRD analysis at *2θ* revealed diffraction peaks (*d*-values) at 8.927, 4.308, 3.490, 2.473, 2.360, 2.220, and 2.043° (Figure 4), which confirmed the unique structure of LRPDNV. The corresponding *2θ* value for *d*-values were 9.900, 20.600, 25.500, 36.300, 38.100, 40.600, and 44.300, respectively, while the relative intensity (I/Io) values of the corresponding d-values were 58, 72, 48, 30, 100, 28, and 44, respectively. An earlier report demonstrated that XRD study of solid lipid nanoparticle for use as an HBsAg delivery system exhibited a laminar lattice [27]. In that study, XRD *2θ* peaks were between 20 to 30° as unique peaks; while shorts peaks were seen at 30 to 40°, and at 40 to 50° [28]. In line with these findings, the present study also exhibited unique *2θ* peaks between 9.900° and 44.300°, thereby demonstrating the crystal lattice structure of the nanovesicles. Interestingly, this study also showed many short peaks (Figure 4), which might be due to the influence of the lyophilization process. However, lyophilization is a very significant process that improves the reconstitution of LRPDNV [29]. The refractivity of LRPDNV was evident in a highly unique pattern from 2 to 8.9°, which indicated the multi-layered crystal lattice formation of nanovesicles. However, decreases in the *2θ* value of nanovesicles indicated different particle sizes in the colloidal injectable dosage form, which was also reflected in the size distribution analysis. Bragg peaks at a relative index of rotation angle of LRPDNV were 58, 72, 48, 30, 100, 28, and 44, consistent with the multilayer crystal laminar structure of nanovesicles [29]. An earlier report suggested that the Bragg peaks at a relative rotation angle of Δ*θ* = 80 indicated a colloidal crystal [29].

#### 3.1.5. NMR Studies

**Figure 5:**
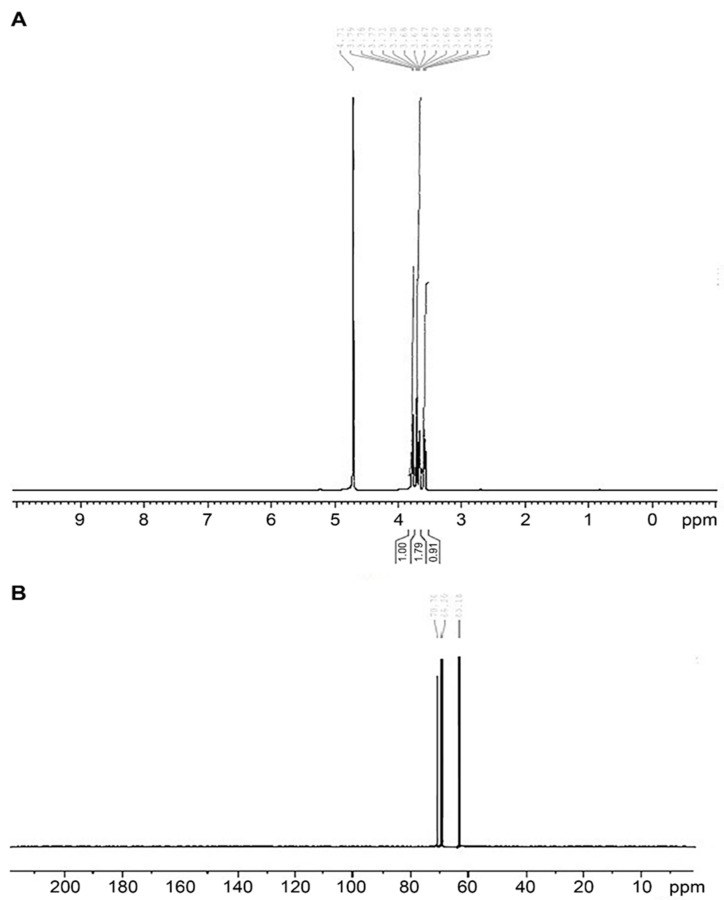
Nuclear magnetic resonance study of lyophilized recombinant HBsAg-loaded docosahexaenoic acid nanovesicles; (**A**) ^1^H-NMR spectrum; (**B**) ^13^C-NMR spectrum.

The ^1^H-NMR analysis gave the fingerprint region of LRPDNV from 0.91 to 1.79 ppm in the proton dimension. The most de-shielded proton peaks appeared at 3. 57, 3. 58, 3.59, 3.60, 3.66, 3.67, 3.67, 3.67, 3. 68, 3.70, 3.71, 3.77, 3.78, 3.79 and 4.71 ppm (Figure 5A). The ppm values are indicative of the presence of fatty acids with a terminal methyl group. The ^1^H-NMR shift from 3.57 to 4.71 ppm indicated the presence of protons from the glycerol moiety of DHA. An earlier study reported that a shift from 5.10–3.70 ppm indicated the presence of protons of the glycerol moiety [30]. The same study suggested that the presence of olefinic protons of unsaturated fatty acids was observed in the region of 5.2–5.5 ppm. However, the present study showed a lack of olefinic protons. The loss of olefinic protons might have occurred during nano vesicular formulation. It has been shown that ^13^C-NMR is a very effective approach for determining the distribution of triacylglycerols and the positions of fatty acids on the glycerol backbone, as well as for providing quantitative information about the fatty acids [31]. The ^13^C-NMR spectrum from 60 to 80 ppm showed the glycerol backbone carbons in DHA (Figure 5B). The present study demonstrated that the nanovesicles were not degraded during formulation and after formulation.

#### 3.1.6. In Vitro Release Profile

**Figure 6:**
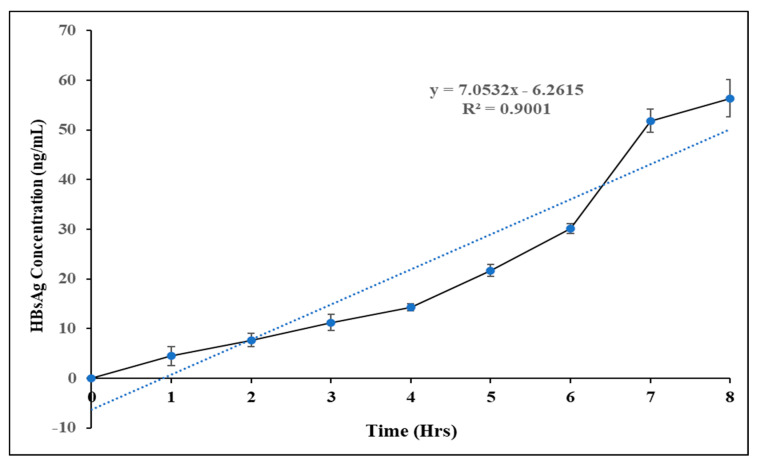
In vitro release profile of HBsAg from lyophilized recombinant HBsAg–loaded docosahexaenoic acid nanovesicles.

The profiles of antigen release under pH 7.4 from HBsAg-loaded LRPDNV are shown in Figure 6. A monophasic release pattern of HBsAg from LRPDNV was observed without initial burst release. The release pattern was first-order kinetics, and the R^2^ value was 0.9, which indicated the linearity of the release profile. The release profile of HBsAg from LRPDNV was much sustained and more or less uniform. The release of HBsAg from LRPDNV into the releasing medium was about 10% in an 8 h study duration, indicating sustained release without any initial burst release. An earlier report showed that the release of HBsAg into PBS was comparatively rapid (approximately 25%) within 16 h [32]. Recently, a study on HBsAg release from three different liposome carriers, solid lipid nanoparticles and nanoparticles reported that 62% release was observed from liposomes, followed by 41% from solid lipid nanoparticles and 29% from polymeric nanoparticles [33]. Moreover, another recent study investigated the release of HBsAg from HBsAg and mannose, which were loaded onto the surface of iron oxide nanoparticles to boost the effectiveness of HBsAg vaccination [34]. The study established that approximately 30% of HBsAg was released on the first day, followed by 60% on the second day [34]. An earlier study demonstrated that the 8–10% HBsAg were released from PLGA, PLA, and chitosan microparticles in 24 h [7]. However, in that study, the release of HBsAg was more in PLGA and PLA microparticles, when compared to chitosan microparticles [7]. According to a previous study, when enteric trimethyl chitosan nanoparticles loaded with HBsAg were administered orally, the amount of HBsAg released from the nanoparticles after 6 h did not exceed 25% of the loaded protein, and 70% of the loaded HBsAg was released after 10 h [35].

### 3.2. In Vivo Study

**Figure 7:**
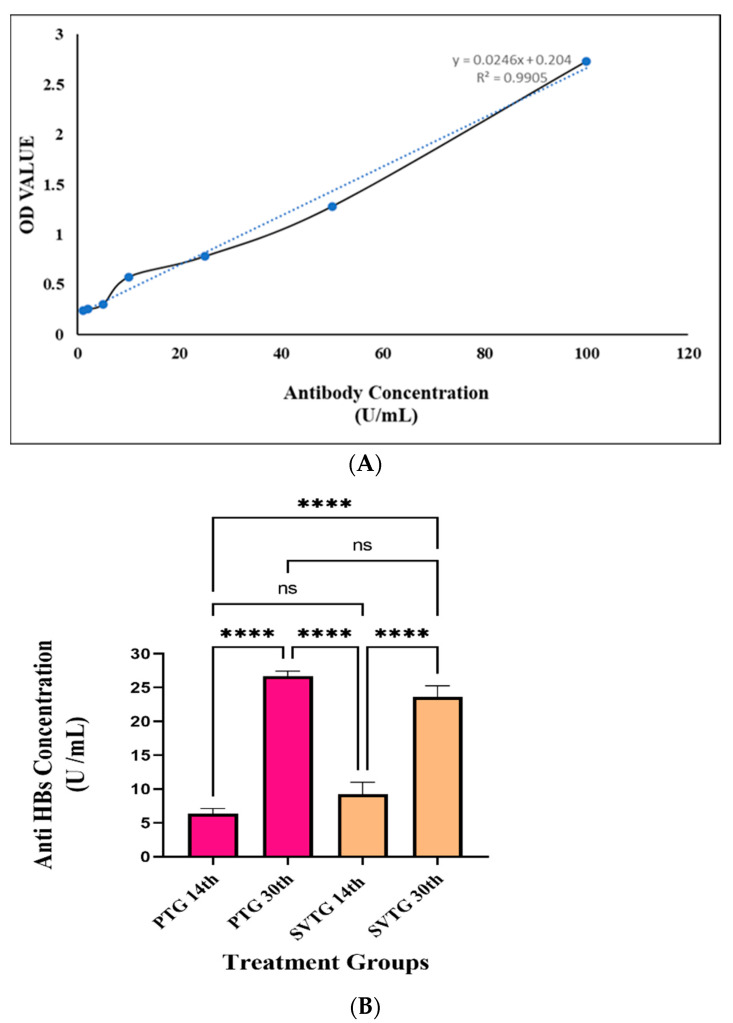
In vivo immunogenicity study; (**A**) standard curve; (**B**) anti-HBs IgG level of various treatment groups. **** The values are very high significant at *p <* 0.05 level, ns: non-significant at *p* < 0.05 level.

Figure 7A depicts the linearity fit of the anti-HBs IgG standard curve, and its R^2^ value is 0.9905. The curve indicated that anti-HBsAg IgG did not degrade. The comparative anti-HBsAg IgG study is shown in Figure 7B. In the product treatment group, the antibody level was 6.35 ± 0.78 U/mL when immunized with LRPDNV on the 14th day. On the other hand, in the standard vaccine treatment group, the specific antibody level was 9.24 ± 1.76 U/mL on the 14th day, which was non-significant at *p* < 0.01 level. Interestingly the antibody level was enhanced after a booster dose both in the product and standard vaccine treatment groups. On the 30th day of the product (LRPDNV) administration, the antibody level was 26.66 0.7 U/mL, which was extremely significant at the *p* < 0.01 level when compared to the antibody level on the 14th day in the product treatment group. The level of antibodies in the product treatment group increased by 318.898% after booster immunization. After booster immunization, the level of antibody in the standard vaccine treatment group was 23.64 U/mL when compared to the 14th day, which was extremely significant at the *p* < 0.01 level. However, following booster immunization, antibody levels increased by 155.844%, which was a lower boosting effect than the product treatment group. The level of antibody in the treatment group was increased by about 12.77% when compared to the standard vaccine on the 30th day. Furthermore, a comparison of antibody levels from the 14th to 30th days showed that the LRPDNV delivery method was successful in releasing antigens over time.

Anti-HBs IgG at a concentration of 10 U/L is necessary to provide protection following immunization [8]. Recombinant HBsAg-loaded chitosan and mannosylated chitosan nanoparticles induced the generation of protective IgG titers [36]. The study demonstrated that mannosylated chitosan nanoparticles have a better adjuvant capacity. An earlier work found that HBsAg-encapsulated methoxy polyethylene glycol-poly(lactide-co-glycolide) nanoparticles significantly induce anti-HBs antibodies [37]. A ferritin nanoparticle vaccine that can transport preS1 to myeloid cells was recently created, resulting in a powerful and long-lasting anti-preS1 response as well as successful viral clearance [38]. The results of this study are self-explanatory and indicate that the nano vesicular system is a more efficient adjuvant for recombinant HBsAg protein than the conventional standard hepatitis B vaccine.

### 3.3. Histopathology Analysis

**Figure 8:**
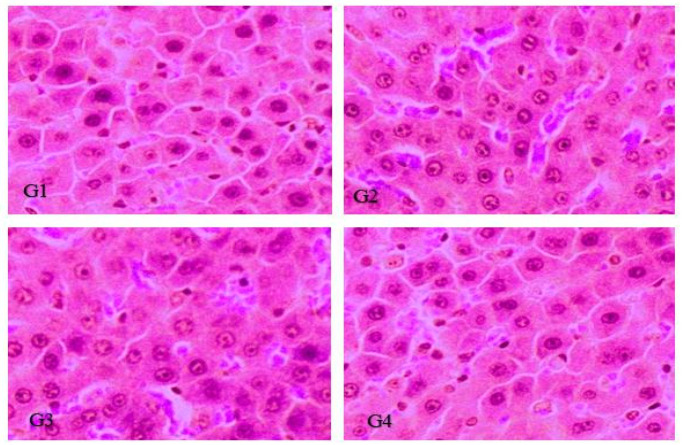
Histopathological examination of the liver by using H & E staining. (**G1**) Normal control without any treatment, (**G2**) treatment with 0.5 mL (product) immunized by IP without significant changes in hepatic cells, (**G3**) treatment with vehicle control 0.5 mL (standard) immunized by IP without any significant changes in hepatic cells, (**G4**) positive control (market standard vaccine) 0.5 mL immunized by IP without any significant changes in hepatic cells.

Figure 8 provides the histopathological changes in different groups of rats. There were no pathological changes observed in group 1. There was also no evidence of any kind of significant hepatic lesion observed in the rest of groups 2, 3, and 4 that indicate that LRPDNV is safe. An earlier study hypothesized that the histological changes created by gold nanoparticles could be an indicator of injured hepatocytes that were unable to deal with the accumulated residues because of the metabolic and structural disruptions caused by these nanoparticles [39]. In contrast, the present study LRPDNV did not produce any histopathological effect on the liver of treated animals since the HBsAg was entrapped in docosahexaenoic acid nanovesicles indicating that the injectable LRPDNV is safer to administer as a vaccine. Interestingly, a previous study revealed that DHA could influence hepatic progenitor cell activation and hepatocyte survival [40].

## 4. Conclusions

In this work, docosahexaenoic acid (DHA) nanovesicles were developed as an adjuvant system for recombinant HBsAg. Based on results from physicochemical characterization, the DHA nanovesicles produced evidence of good potential as a promising adjuvant system for recombinant HBsAg. These findings suggest that the DHA nanovesicles are very compatible with recombinant proteins. Furthermore, recombinant HBsAg entrapped DHA nano vesicles induced high-level anti-HBs IgG in Wistar rats. Overall, the present study suggested that the DHA nanovesicles are a better adjuvant for recombinant HBsAg protein. Thus, they can be used as a delivery system for recombinant proteins.

## Figures and Tables

**Table 1:** Physical characterization of lyophilized recombinant HBsAg-loaded docosahexaenoic acid nanovesicles.

Zeta Potential (mV)	PDI	% PDI	Particle Size (z. d.nm)	Particle Size in Mass (r.nm)	% Mass r.nm	Mobility (µm cm/Vs)	Conductivity (mS/cm)
−60.4 ± 10.4	0.201	74.8	361.4 ± 48.24	298.8 ± 13.4	50	−3.417	0.728

## Data Availability

The data used to support the findings of this study are included within the article.
